# Acute Lymphoblastic Leukemia in Routine Practice: A Turkish Multicenter Study

**DOI:** 10.4274/tjh.galenos.2019.2019.0008

**Published:** 2019-08-02

**Authors:** Rafiye Çiftçiler, Ömür Gökmen Sevindik, Ali İrfan Emre Tekgündüz, Mehmet Ali Erkurt, Filiz Vural, Burhan Turgut, Leylagül Kaynar, Bahriye Payzın, Mehmet Hilmi Doğu, Volkan Karakuş, Fevzi Altuntaş, Yahya Büyükaşık, Fatih Demirkan

**Affiliations:** 1 Hacettepe University Faculty of Medicine, Department of Hematology, Ankara, Turkey; 2 Medipol University Hospital, Clinic of Hematology, İstanbul, Turkey; 3 Memorial Bahçelievler Hospital, Clinic of Hematology, İstanbul, Turkey; 4 İnönü University Faculty of Medicine, Department of Hematology, Malatya, Turkey; 5 Ege University Faculty of Medicine, Department of Hematology, İzmir, Turkey; 6 Namık Kemal University Faculty of Medicine, Department of Hematology, Tekirdağ, Turkey; 7 Erciyes University Faculty of Medicine, Department of Hematology, Kayseri, Turkey; 8 İzmir Atatürk Training and Research Hospital, Clinic of Hematology, İzmir, Turkey; 9 İstanbul Training and Research Hospital, Clinic of Hematology, İstanbul, Turkey; 10 Muğla Sıtkı Koçman University Faculty of Medicine, Department of Hematology, Muğla, Turkey; 11 Ankara Oncology Training and Research Hospital, Clinic of Hematology, Ankara, Turkey; 12 Dokuz Eylül University Faculty of Medicine, Department of Hematology, İzmir, Turkey

**Keywords:** Acute lymphoblastic leukemia, Pediatric regimen, Pediatric-inspired regimen, Philadelphia chromosome

## Abstract

**Objective:**

Significant developments occurred in the clinical management of acute lymphoblastic leukemia (ALL) in adults in recent decades. However, treatment results are still not satisfactory, especially in routine practice. The objective of this study was to evaluate the general clinical features, treatment details, and outcomes of a large group of patients followed in multiple centers in Turkey with a diagnosis of ALL.

**Materials and Methods:**

A retrospective analysis of the data of patients with ALL was made, the patients having been diagnosed and treated between January 2003 and June 2017 by different protocols in the hematology clinics of ten different centers. A total of 288 patients, aged between 17 and 76 years old, were included in the study. In this retrospective multicenter analysis of patients with ALL, classification of patients was performed based on treatment period, Philadelphia chromosome positivity, treatment regimen, and administration of allogeneic hematopoietic stem cell transplantation (allo-HSCT).

**Results:**

The majority of cases were B-cell in origin, while 224 patients had B-ALL and 64 of the patients had T-ALL. Median follow-up duration for all patients was 18.2 months (range: 0.03-161 months). Philadelphia chromosome positivity was determined in 49 patients (21.9%), and 54 patients (18.8%) were receiving allo-HSCT. After induction chemotherapy, 219 patients (76.0%) achieved complete remission, 32 patients (11.2%) were evaluated as treatment refractory, and 37 patients (12.8%) were deceased. Median overall survival was 47.7 months (95% confidence interval: 36.1-59.2) and median disease-free survival was 23.4 months (95% confidence interval: 6.7-40.0) for all patients.

**Conclusion:**

Multicenter studies are extremely important for defining the specific clinical features of a particular disease. The results of this study will make a significant contribution to the literature as they reflect real-life data providing valuable information about the Turkish ALL patient profile.

## Abstract (tr)

**Amaç:**

Son yıllarda erişkinlerde akut lenfoblastik löseminin (ALL) tedavi yönetiminde önemli gelişmeler meydana gelmiştir. Bununla birlikte, özellikle rutin uygulamada tedavi sonuçları hala tatmin edici değildir. Çalışmanın amacı, Türkiye’de birden fazla merkezde takip edilen geniş bir ALL hasta grubunun genel klinik özelliklerini, tedavi özelliklerini ve sağkalım sonuçlarını değerlendirmektir.

**Gereç ve Yöntemler:**

Ocak 2003 ile Haziran 2017 tarihleri arasında on farklı merkezdeki hematoloji kliniğinde tanı konulan ve farklı protokollerle tedavi edilen ALL hastalarının verilerinin retrospektif analizi yapıldı. Çalışmaya yaşları 17 ile 76 arasında değişen toplam 288 hasta dahil edildi. Bu çalışmada ALL’li hastalar tedavi periyoduna, Philadelphia kromozom pozitifliğine, tedavi protokolü ve allojenik hematopoetik kök hücre nakli (AHKHN) yapılıp yapılmamasına göre sınıflandırıldı.

**Bulgular:**

Olguların 224’ü B-ALL, 64’ü T-ALL idi. Tüm hastalar için ortanca takip süresi 18,2 ay (dağılım, 0,03-161 ay) idi. Philadelphia kromozomu (Ph+) pozitifliği 49 (%21,9) hastada saptandı ve 54 hastaya (%18,8) AHKHN yapıldığı izlendi. İndüksiyon kemoterapisinden sonra 219 hasta (%76) tam remisyona girdi, 32 hasta tedaviye refrakter (%11,2) olarak değerlendirildi, 37 hastada ise (%12,8) mortalite gözlendi. Ortanca genel sağkalım 47,7 ay (%95 güven aralığı: 36,1-59,2) ve ortanca hastalıksız sağkalım tüm hastalar için 23,4 ay (%95 güven aralığı: 6,7-40,0) idi.

**Sonuç:**

Sonuç olarak, çok merkezli çalışmalar, belirli bir hastalığın spesifik klinik özelliklerini tanımlamak için büyük öneme sahiptir. Bu çalışmanın sonuçları, Türk ALL hasta profili hakkında değerli bilgiler sağlayan gerçek hayat verilerini yansıttığı için literatüre önemli bir katkı sağlayacaktır.

## Introduction

Significant developments have occurred in the clinical management of acute lymphoblastic leukemia (ALL) in adults in the last decades. However, treatment results are still not satisfactory, especially in routine practice. Disease biology, higher-risk leukemia genetics, absence of sufficiently effective therapies, noncompliance with treatment, and intolerance of chemotherapy are the main reasons for unsatisfactory results [1]. Another challenge in adult ALL treatment is the lack of a standardized regimen. However, gradual stable improvement has been observed recently in adult ALL as a result of adopting pediatric-inspired regimens, better identification of high-risk patients and early referral for allogeneic hematopoietic stem cell transplantation (allo-HSCT), increased availability of donors, and the inclusion of minimal residual disease (MRD) in ALL treatment decisions [2,3]. The objective of this study was to evaluate the general clinical features, treatment details, and outcomes of a large group of patients with ALL followed in multiple centers in Turkey.

## Materials and Methods

### Study Design and Data Collection

A retrospective analysis was made of the data of ALL patients who were diagnosed and treated with different protocols in ten hematology clinics between January 2003 and June 2017. Patients were classified by treatment period, Philadelphia chromosome positivity (Ph+), treatment regimen, and administration of allo-HSCT. A total of 288 patients were included from the ten participating centers. The primary outcome was overall survival (OS) and secondary outcomes were complete remission (CR) rate and disease-free survival (DFS).

The presence of ALL was diagnosed by the detection of ≥20% blasts in the bone marrow. Immunophenotype was detected using flow cytometry (peripheral blood or bone marrow) or immunohistochemical techniques (bone marrow biopsy or aspiration) for each patient. The presence of the Ph chromosome/BCR-ABL fusion transcript was investigated using conventional cytogenetic analysis (karyotyping) on bone marrow samples. Quantitative polymerase chain reaction (RQ-PCR) was performed to monitor BCR/ABL fusion transcripts during follow-up.

Intensive regimens were classified as pediatric regimens (BFM, Dana Farber), pediatric-inspired chemotherapy (DFCI-ALL consortium protocol, CALGB, Linker-4 regimen, MRC UK-ALLXII/ECOG-2993, GMALL), or adult intensive chemotherapy (HYPERCVAD, CODOX-M, CHOEP, GRAALL-2003, FLAG, FLAG-IDA). Nonintensive regimens were classified as POMP and EWALL regimens. A total of 86 patients (29.9%) were treated with a pediatric regimen, 84 (29.1%) with pediatric-inspired chemotherapy, 105 (36.5%) with adult intensive chemotherapy, and 13 (4.5%) with a nonintensive regimen. At the time of diagnosis, patients with good general condition and good ECOG performance status were treated with intensive regimens such as a pediatric regimen, pediatric-inspired chemotherapy, or adult intensive chemotherapy. Patients with poor general condition and poor ECOG performance status were given nonintensive regimens such as EWALL and POMP chemotherapy protocols. The treatment regimens are presented in Table 1.

All patients received growth factor support during chemotherapy, and anti-infective prophylaxis with fluconazole (400 mg/day), valacyclovir (2x500 mg/day), and trimethoprim/sulfamethoxazole (160/800 mg, 2 days a week) was used in the intensive chemotherapy group. All patients underwent bone marrow aspiration and biopsy on median day 28 of treatment (range: days 18-48) to evaluate patients’ responses to induction chemotherapy. CR was defined as <5% blasts in regenerating bone marrow without the finding of extramedullary residual disease.

All of the ethical considerations were strictly followed in accordance with the 1964 Helsinki Declaration. As standard care/action of the hospitals has been recognized from the patient records that all of the studied patients gave informed consent at the time of admission to the hospital and before the administration of chemotherapy and other relevant diagnostic/therapeutic standards of care.

### Statistical Analysis

Demographic characteristics were presented using proportions and medians (minimum-maximum) for categorical and continuous variables, respectively. Statistical comparisons were made using the chi-square test for categorical data. Survival analyses were made using the Kaplan-Meier test. The log-rank test was applied to compare survival data. OS was calculated from the date of diagnosis to death for any reason. Surviving patients were counted on the date of the final follow-up examination. DFS was calculated from the date of CR to relapse or death in remission. Patients surviving in remission were counted on the date of the final follow-up examination. Univariate analyses of the differences in OS and DFS were applied using log-rank tests. Receiving allo-HSCT, age (≤30 years), sex (male), Ph chromosome (negativity), treatment regimen (pediatric regimen vs. other regimens), and time period (2011-2017 vs. 2003-2011) were evaluated as prognostic factors. Univariate comparisons with a p-value of <0.15 were included in the multivariate analyses, in which p<0.05 was considered statistically significant. Cox regression analysis was performed to study the simultaneous impact of selected factors on survival. Values of p<0.05 were accepted as statistically significant. The statistical analyses were conducted using SPSS 17 (SPSS Inc., Chicago, IL, USA).

## Results

### Patient and Treatment Group Characteristics

The study included 288 patients, consisting of 173 males and 115 females with a median age of 34 (range: 17-95 years). The majority of cases were B-cell in origin; 224 (77.8%) patients had B-ALL and 64 (22.2%) had T-ALL. Philadelphia chromosome positivity (Ph+) was determined in 49 (21.9%) patients. All patients with Ph+ ALL were diagnosed after 2005. Imatinib was added to the chemotherapy protocol for 47 Ph+ ALL patients (95.9%). Rituximab was added to the chemotherapy protocol for patients with CD20-positive B-cell ALL. Allo-HSCT was administered to 54 (18.8%) patients. The clinical characteristics of the ALL patients are given in Table 2.

### Overall Outcomes

The median follow-up period for all patients was 18.2 months (range: 0.03-161.0 months). After induction chemotherapy, 219 patients (76.0%) achieved complete remission, 32 patients (11.2%) were evaluated as treatment refractory, and 37 patients (12.8%) were deceased. Median OS was 47.7 months (95% confidence interval (CI): 36.1-59.2) and median DFS was 23.4 months (95% CI: 6.7-40.0) for all patients. The 3-year OS and DFS rates were 56% and 45%, respectively. The 5-year OS and DFS rates were 43% and 35%, respectively.

**Figure 1:**
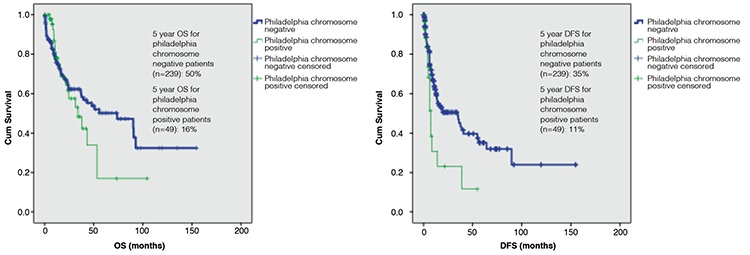
Overall survival (p=0.48) and disease-free survival (p=0.008) for acute lymphoblastic leukemia patients according to Philadelphia chromosome status. OS: Overall survival, DFS: disease-free survival.

Median OS was 33.9 months (95% CI: 17.4-50.3) for Ph+ ALL patients and 73.7 months (95% CI: 33.9-113.5) for Ph-negative (Ph-) ALL patients (p=0.48). Median DFS was 7.1 months (95% CI: 5.0-9.3) for Ph+ ALL patients and 34.6 months (95% CI: 16.0-53.2) for Ph- ALL patients. DFS was statistically significant longer in Ph- patients than Ph+ patients (p=0.008). The 5-year OS was 50% in Ph- patients and 16% in Ph+ patients, respectively. The 5-year DFS was 35% in Ph- patients and 11% in Ph+ patients (Figure 1).

**Figure 2:**
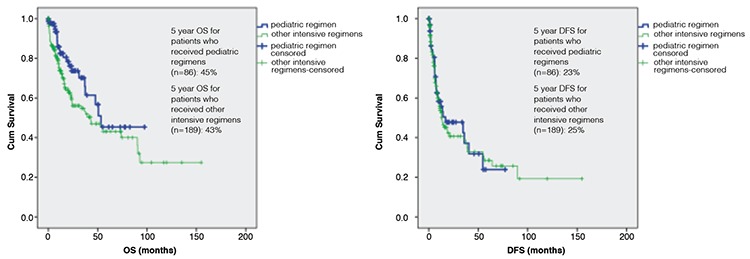
Overall survival (p=0.05) and disease-free survival (p=0.78) for acute lymphoblastic leukemia patients according to pediatric regimens and other intensive regimens. OS: Overall survival, DFS: disease-free survival.

Median OS was 53.4 months (95% CI: 37.9-63.5) in patients receiving a pediatric regimen and 42.9 months (95% CI: 19.9-66.6) in patients receiving other intensive regimens (p=0.05). Median DFS was 16.9 months (95% CI: 3.9-23.6) in patients receiving a pediatric regimen and 13.3 months (95% CI: 6.5-20) in patients receiving other intensive regimens (p=0.78). The 5-year OS was 45% in patients who received a pediatric regimen and 43% in patients who received other intensive regimens. The 5-year DFS was 23% in patients who received a pediatric regimen and 25% in patients who received other intensive regimens (Figure 2).

When only the patients under 30 years of age were analyzed, no significant difference was determined between pediatric and other intensive regimens in terms of OS and DFS. The 3-year OS was 77% in patients under 30 years of age who received a pediatric regimen and 66% in patients who received other intensive regimens. The 5-year OS was 53% in patients under 30 years of age who received a pediatric regimen and 56% in patients who received other intensive regimens (p=0.68). The 3-year DFS was 54% in patients under 30 years of age who received a pediatric regimen and 45% in patients who received other intensive regimens. The 5-year DFS was 36% in patients under 30 years of age who received a pediatric regimen and 31% in patients who received other intensive regimens (p=0.84).

**Figure 3:**
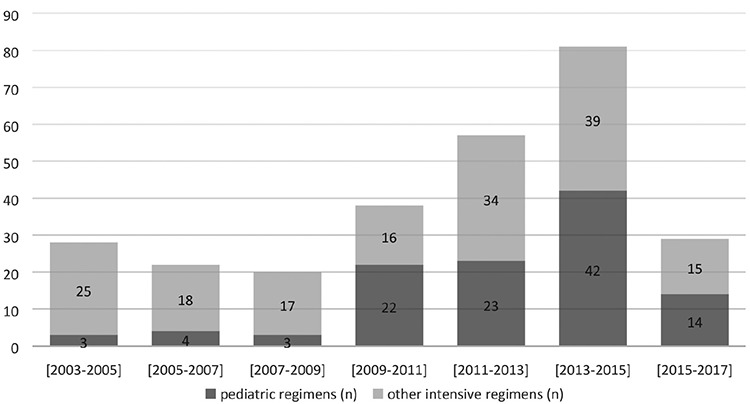
Two-year periods between 2003 and 2017 for treatment regimens.

The 2-year periods between 2003 and 2017 for treatment regimens are shown in Figure 3. From 2003 to 2017, the usage of pediatric regimens increased in ALL patients. The count of patients diagnosed with ALL and the treatment protocols used were observed to vary over time. While adult intensive regimens were used more commonly in the past, there is a tendency to use pediatric regimens at present time.

The follow-up dates between January 2003 and April 2017 were divided into 7 treatment periods (Table 3). There was no statistically significant improvement in OS according to time among the ALL patients (p=0.58). There was no statistically significant improvement in DFS according to time for ALL patients (p=0.92). The success of CR did not differ significantly over the years (p=0.49). OS and DFS were higher between the years of 2007 and 2011 compared to other periods. OS and DFS were higher in the 2007-2011 period, but not at a statistically significant level. The 5-year OS rate was 73% in 2007-2011 and 36% in the remaining period (p=0.08). The 5-year DFS rate was 55% in 2007-2011 and 24% in the remaining period (p=0.11).

### Cox Regression Analyses

In univariate analyses the variables that affected OS were determined to be receiving allo-HSCT (p=0.01), cellular origin of the disease (B-cell ALL) (p=0.02), patient age (≤30 years) (p=0.001), being male (p=0.03), and treatment regimen (receiving a pediatric regimen) (p=0.05) (Table 4). Cox regression analysis revealed patient age (≤30 years) (p<0.001) and cellular origin of the disease (B-cell origin) (p=0.04) as the parameters predicting OS.

In univariate analyses the variables that affected DFS were receiving allo-HSCT (p=0.02), patient age (≤30 years) (p=0.02), being male (p=0.10), and Ph chromosome negativity (p=0.009). Cox regression analysis did not reveal any parameters predicting DFS.

## Discussion

ALL is one of the most common hematological malignancies in pediatric patients, with a cure rate of approximately 80%. However, in adults, ALL is uncommon and has a bad prognosis [9]. Despite the improvement in CR rates, most adults with ALL will relapse and finally die because of this illness. Although advanced therapy such as allo-HSCT and supportive care for ALL patients has improved the OS rate, unfortunately it is still as poor as 30%-40% at 5 years [18,19,20].

In this study, patients were classified by treatment period, Philadelphia chromosome positivity, treatment regimen, and administration of allo-HSCT. The results consisted of induction therapy and survival outcomes of patients from ten participating centers. A variety of factors were determined that could affect OS and DFS in adult ALL patients, such as Philadelphia chromosome negativity, treatment regimen, younger age, being male, cellular origin of the disease, and receiving allo-HSCT.

Ph+ ALL is a leukemia mainly encountered in adult patients, and its incidence tends to grow with age [21]. The Ph chromosome marks a group of patients at very high risk, as shown by the data published by a French study in which of 25 patients with Ph+ ALL only 60% reached CR after a course of chemotherapy and 4 patients reached CR after salvage chemotherapy. The CR rate was 76%, the mean DFS was 5.6 months, and the mean OS was 10.1 months [22]. Gokbuget et al. [23] reported 50 Ph+ patients who had poor responses to age-adjusted chemotherapy compared to Ph- patients. The remission rate was reported as 19% for Ph+ patients and 64% for Ph- patients. In the current study, OS was longer in Ph- patients than Ph+ patients but there was no statistically significant difference. In addition, DFS was statistically significantly longer in Ph- patients than Ph+ patients. All patients with Ph+ ALL were diagnosed after 2005 in this study. Imatinib treatment was added to the treatment protocols of 95.9% of patients with Ph+ ALL. Tyrosine kinase inhibitors are added in most standard ALL protocols for young patients with Ph+ ALL. However, for older patients higher induction mortality rates have been reported with such approaches [24,25]. Some studies that examined the use of a combination of tyrosine kinase inhibitors (imatinib or dasatinib) with mild therapy including a steroid with or without vincristine reported CR in the majority of patients with Ph+ ALL [26,27].

Significant improvements have been achieved in ALL treatment using new clinical protocols. Developments regarding the immunobiology of ALL and better recognition of prognostic factors have resulted in better characterization of risk groups and the tailoring of therapy [4,5,28]. In the current study, the follow-up period between January 2003 and June 2017 was divided into 7 treatment periods. There was no statistically significant improvement in survival outcomes in ALL patients according to these periods. Furthermore, the achievement of CR was not determined to have been improved over time in the overall study period. It is thought that this is due to the fact that new agents are not added to treatment protocols for the treatment of ALL in adults, especially in developing countries. Recent developments in the treatment of ALL need to be used more effectively. Additionally, every ALL patient should be followed with MRD. Treatment options after induction chemotherapy of patients, especially allo-HSCT, should be planned according to the results of MRD follow-up, but unfortunately very few centers in Turkey follow MRD.

Over the recent 40 years, numerous efficacious chemotherapy regimens have been developed for the treatment of ALL, mostly based on pediatric regimens. Some prospective trials have compared different regimens in ALL patients. Most progress in ALL treatment has been seen in children and adolescents, while the advancement reported for adults is restricted [29,30]. The existing data in terms of tolerability and efficacy of BFM chemotherapy in adults are insufficient [31,32]. In a single-center study with BFM chemotherapy in adult ALL patients, the CR rate was 90% with induction chemotherapy and 5-year OS was 62% [32]. The current study showed that there was a nearly statistically significant difference in OS in patients receiving a pediatric regimen compared to other intensive regimens (p=0.05). Furthermore, there was no statistically significant difference in DFS in patients receiving the original pediatric regimen compared to other intensive regimens (p=0.78). Pediatric regimens did not show a substantial superiority in this study. As in children, the choice is sometimes made to not give optimal dose treatment to adult ALL patients, considering that the patients cannot tolerate it. Therefore, pediatric regimens may not have demonstrated superiority in this study. We think that new agents and new protocols should be considered in the treatment of adult ALL patients.

Allo-HSCT remains an important component in adult ALL therapy, and it is particularly demonstrated to be a main part of treatment for adults with high-risk ALL [1]. In the univariate analysis of the current study, OS and DFS were significantly higher in patients who underwent allo-HSCT. However, only 18.7% of the total 288 patients received allo-HSCT. In both univariate and multivariate analysis, OS was significantly higher in young patients (≤30 years) than in patients aged >30 years.

Previous multiple retrospective analyses in adult and pediatric prospective clinical studies comparing the outcomes of ALL treatment in adolescents and young adults have demonstrated the superiority of pediatric regimens in those age groups over adult regimens [33,34]. In the current study, the superiority of the pediatric regimens in terms of both OS and DFS could not be demonstrated compared to other intensive regimens. OS was almost statistically significantly better in patients who received pediatric regimens compared to patients who received other intensive regimens, such as pediatric-inspired chemotherapy and adult intensive regimens. In the univariate analyses, however, there was no statistically significant improvement in OS or DFS according to time in ALL patients. DFS was statistically significantly higher in Ph- patients than Ph+ patients in univariate analyses. However, this was not statistically significant in multivariate analyses. The outcomes of adults with T-cell ALL have improved with recent ALL regimens, and outcomes today are comparable to those of patients with the precursor B-cell subtype [35,36]. In this study, patients with B-cell ALL had longer OS than patients with T-cell ALL in univariate and multivariate analyses. However, DFS was not affected by the cellular origin of the disease.

### Study Limitations

This study had some limitations. First, the study was retrospective, and second, cytogenetic results were not obtained for each patient. Third, there were differences in treatment modalities between the centers, so many different types of treatment regimens were used for these ALL patients. Moreover, the infrastructure and quality of each center are different. Hence, it is very difficult to draw conclusions from these heterogeneous results. Searching for an improvement in CR rates in such a heterogeneous group of patients is highly challenging. The retrospective nature and treatment heterogeneity may be a limitation; however, at the same time they are the major strengths of this study. In addition, outside of clinical studies, pediatric protocols may fail to substantially improve outcomes due to excessive toxicity, treatment delays, and dose reductions.

## Conclusion

Multicenter studies are particularly important for defining the specific clinical features of a particular disease. The results of this study can be considered to make a significant contribution to the literature because they reflect real-life data providing valuable information about the Turkish ALL patient profile.

## Figures and Tables

**Table 1:**
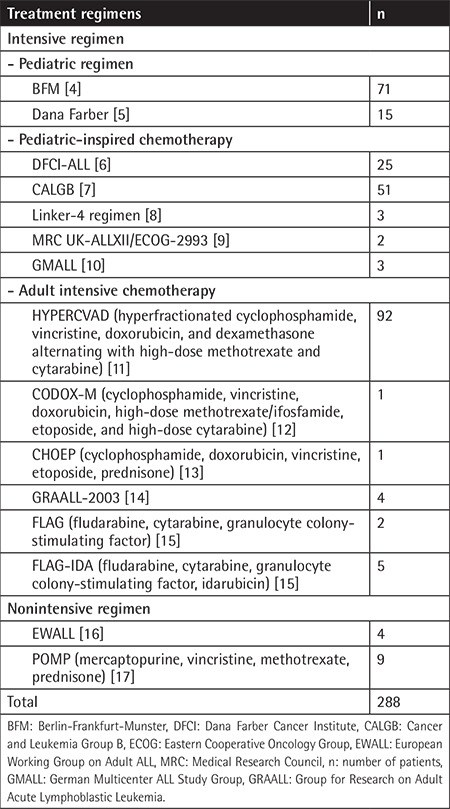
Treatment regimens.

**Table 2:**
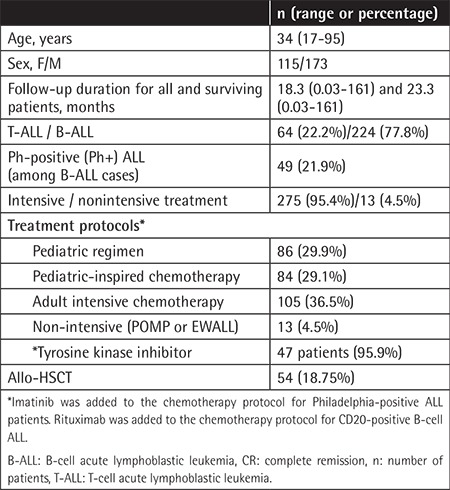
Baseline characteristics of acute lymphoblastic leukemia patients.

**Table 3:**
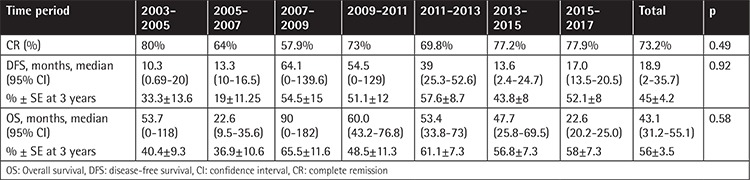
Complete remission rate, overall survival, and disease-free survival according to time periods.

**Table 4:**
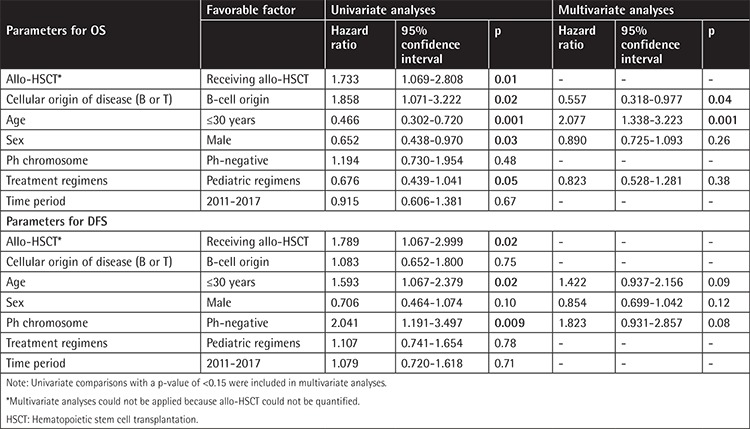
Univariate and multivariate analyses (Cox model) of overall survival and disease-free survival.
